# Spatial–Numerical and Ordinal Positional Associations Coexist in Parallel

**DOI:** 10.3389/fpsyg.2016.00438

**Published:** 2016-03-30

**Authors:** Stefan Huber, Elise Klein, Korbinian Moeller, Klaus Willmes

**Affiliations:** ^1^Leibniz-Institut für WissensmedienTübingen, Germany; ^2^Eberhardt-Karls University TübingenTübingen, Germany; ^3^Section Neuropsychology, Department of Neurology, University Hospital, RWTH Aachen UniversityAachen, Germany

**Keywords:** SNARC effect, ordinal position effect, number processing, parity task, number–space association

## Abstract

There is evidence for a systematic association of numbers and space. A prominent finding supporting this notion is the spatial-numerical association of response codes (SNARC) effect describing relatively faster responses to smaller numbers using a left-hand key and to larger numbers using a right-hand key. However, the assumption of the SNARC effect reflecting spatial-numerical associations was challenged recently. A working memory account was proposed suggesting that not numbers per se but their position in a memorized sequence is associated with space. Yet, there is also first evidence suggesting that this ordinal position and the SNARC effect may not be mutually exclusive. In the present study, we further examined the relationship between the ordinal position and the SNARC effect. We manipulated the number of items in the memorized ordered sequence and the number range employed. Results revealed both a significant ordinal position effect, but also a significant SNARC effect, substantiating the view that both effects are not mutually exclusive but may co-exist. Furthermore, we found that the SNARC effect was reduced when numbers ranging from 1 to 10 versus numbers ranging from 1 to 9 were employed. Thus, our results question a pure working memory account for the SNARC effect. Additionally, they highlight the critical role of the number range employed in research about the SNARC effect.

## Introduction

In numerical cognition research there is the notion of a specific association of the representations of numbers and space. Such an association was already described by Galton, who reported that some individuals explicitly represent numbers in a spatially organized manner, which he termed “number forms” (Galton, 1880; see also Seron et al., 1992). A more recent finding supporting the association between numbers and space came from Dehaene et al. (1993). The authors investigated response time (RT) differences between left hand and right hand responses when judging the parity of numbers. Although the magnitude of the numbers is completely irrelevant in the parity judgment task, Dehaene et al. (1993) observed that for relatively smaller numbers left hand responses were faster, whereas for relatively larger numbers right hand responses were faster. The authors interpreted this pattern of results to suggest not only automatic processing of task-irrelevant number magnitude, but also to reflect a spatially oriented representation of number magnitude often described by the metaphor of a mental number line (MNL), upon which numbers are internally represented in ascending order from left to right. Therefore, small numbers are associated with “left” and large numbers with “right”, driving the observed *spatial numerical association of response codes* or, in short, the SNARC effect (visuospatial account). The SNARC effect has been replicated repeatedly in various studies employing different task settings and populations (see Wood et al., 2008, for a meta-analysis). However, there is also evidence that the association between numbers and space (as indicated by the SNARC effect) is flexible rather than hard-wired (e.g., Fischer and Brugger, 2011).

First evidence for this suggestion was again provided by Dehaene et al. (1993). The authors found that the RT advantage of left vs. right hand responses for relatively smaller numbers did not depend on the absolute magnitude of numbers, but on the relative magnitude of the respective numbers (see also Fias et al., 1996). Depending on the number range assessed, the numbers 4 or 5 can be perceived as either small (in case of a number range from 4 to 9) or large (in case of a number range from 1 to 5). Furthermore, when priming participants with clock-faces, on which small numbers are located on the right, participants responded faster to small numbers with the right hand and to large numbers with the left hand (Bächtold et al., 1998). And even reading a cooking recipe containing numbers on either the left or right in the text modulated the association between numbers and space as indicated by the SNARC effect (Fischer et al., 2010).

To explain these findings, a verbal-spatial account was proposed (Proctor and Cho, 2006; Gevers et al., 2010) suggesting that the SNARC effect arises from associations between the verbal concepts “small”/“large” and “left”/“right”. The verbal–spatial account can, for instance, explain the reversed SNARC effect when priming participants with clock-faces (Bächtold et al., 1998). In this condition, participants associated the verbal concepts “small” with “right” and “large” with “left” according to the position of these numbers on a standard clock-face resulting in a reversed SNARC effect.

More recently, van Dijck and Fias (2011) suggested a new account proposing the SNARC effect to result from a temporary association of numbers and space to be formed in working memory, rather than reflecting a long-term memory MNL representation (Herrera et al., 2008; van Dijck et al., 2009; van Dijck and Fias, 2011; Ginsburg et al., 2014). In line with this view, Herrera et al. (2008) and van Dijck et al. (2009) found a reduced SNARC effect under working memory load. Picking up on these findings, van Dijck and Fias (2011) examined in what way working memory might account for the SNARC effect. In their study, participants had to remember a sequence of five numbers (taken from the range 1–10). Then further numbers were presented and participants had to judge the parity (by pressing a respective response button) of those numbers included in the to-be-memorized sequence only. Finally, participants had to single out the to-be-memorized sequence out of five alternatives. Interestingly, using this paradigm the authors did not find a regular SNARC effect. Instead, they observed that the position of numbers in the to-be-memorized sequence was associated with space. Participants responded faster with the left hand than the right hand to numbers, which were presented early in the to-be-memorized sequence, whereas numbers presented toward the end of the sequence were responded to faster with the right hand (*ordinal position effect*). Accordingly, van Dijck and Fias (2011) concluded that the SNARC results from a temporary association of the ordinal position of numbers and space to be formed in working memory. Thus, they suggested that in a regular parity judgment task participants “make use of the inherent ordinal structure of the number system and systematically map numbers to the temporary task-set store as a function of numerical magnitude.” (Ginsburg et al., 2014, p. 9)

However, this conclusion is hard to reconcile with the results of Lindemann et al. (2008). Similar to van Dijck and Fias (2011), they used a paradigm with a memorization phase (memorization of a sequence of three digits) and a classification phase (classification of digits according to their parity status). However, different from van Dijck and Fias (2011), the authors manipulated the sequence type and used sequences of ascending (e.g., 3–4–5), descending (e.g., 5–4–3) or no specific order (e.g., 5–3–4). Lindemann et al. (2008) found that the SNARC effect disappeared only in the condition with descending order. To explain this finding, they suggested that the descending coding in working memory and the spatial representation of numbers interfered with each other in this condition, resulting in the absence of the SNARC effect. Thus, this study indicates that both long-term memory associations between numbers and space as well as temporarily established associations between the sequential position of numbers and space formed in verbal working memory coexist. However, they can interfere and may even cancel out each other in case of descending sequences.

Further evidence for the coexistence of temporary associations in working memory and long-term memory associations was provided by Ginsburg and Gevers (2015). In their study, the authors used a similar paradigm as van Dijck and Fias (2011) with an encoding phase (memorization of a sequence of numbers), a classification phase (classifying numbers as smaller or larger than 5) and a control phase (judging whether a presented sequence is identical to the memorized sequence). However, different from van Dijck and Fias (2011), they used a task-switching paradigm in the classification phase. The task-switching paradigm was established by dividing the experiment in different blocks. At the beginning of each block, participants were given a task cue indicating whether they had to respond to all numbers (inducer task) or only to numbers in the memorized sequence (diagnostic task). Interestingly, Ginsburg and Gevers (2015) observed a SNARC effect as well as an ordinal position effect in the diagnostic task only. As the effects did not interact, they interpreted this finding to suggest that the SNARC effect and the ordinal position effect indicate the activation of different representations: long-term memory associations between numbers and space in case of the SNARC effect and temporarily established associations between the ordinal position of numbers and space formed in verbal working memory.

In the present study, we further examined the relationship between the SNARC and the ordinal position effect. In particular, we were interested why van Dijck and Fias (2011) as well as Ginsburg et al. (2014) did not observe a signifant SNARC effect, although Lindemann et al. (2008) provided evidence that the SNARC effect diminishes only in case of descending order of numbers in the to-be-memorized sequence. van Dijck and Fias (2011) as well as Ginsburg et al. (2014) used random sequences of five numbers ranging from 1 to 10. Thus, they did not specify the ordering of the sequences. To get an impression how many of their sequences might have been in descending order, we ran a simulation with 100,000 runs randomly drawing 5 numbers ranging from 1 to 10 and categorized them as representing an either ascending (e.g., 2–5–6–8–10), descending (e.g., 10–8–6–5–2) or no specific order (e.g., 5–4–1–9–3). We found that less than 1% of all sequences were in descending order. Even when we considered partially descending sequences (i.e., at least three numbers in sequence are in descending order; e.g., 6–5–2–10–1), only 31.9% would have been in partially descending order. In turn, most of the sequence were either in ascending (0.9%), partially ascending (40.6%) or in no specific order (26.7%) and thus, a regular SNARC effect should have been expected. Therefore, ordering cannot explain the differing results of van Dijck and Fias (2011) and Ginsburg et al. (2014) exclusively. However, an explanation for the absence of a SNARC effect might be the longer sequence length of five numbers, because participants might override the canonical number coding in working memory depending on the working memory load.

Another difference to the study of Lindemann et al. (2008) is that van Dijck and Fias (2011) as well as Ginsburg et al. (2014) included the number “10” in the stimulus set. Including 10 might reduce or even eliminate the numerical SNARC effect. Dehaene et al. (1993) investigated, inter alia, whether the SNARC effect may also be found for the number range from 10 to 19. However, they did not observe a significant SNARC effect for the number range 10 to 19. Interestingly, the leading digit 1 seemed to interfere with the “even” response and rather facilitated “odd” responses suggesting that the parity of both digits constituting two-digit numbers may be processed separately and in parallel (Dehaene et al., 1993; Tan and Dixon, 2011; Huber et al., 2015). Additionally, the number 10 is not only problematic because it is a two-digit number, but also because it contains a zero at the unit position. Participants are often not sure about the parity status of zero (Nuerk et al., 2004). Moreover, Nuerk et al. (2004) also found that the parity status of zero is somewhat distinct from even and odd digits by employing a non-metric multidimensional scaling procedure to depict the pattern of RT intercorrelations among the numbers presented.

These two potential explanations for the absence of the SNARC effect in the studies of van Dijck and Fias (2011) as well as Ginsburg et al. (2014) were investigated in the present study. As most of the sequences in the respective studies might have been unordered sequences, we also used unordered sequences, but we manipulated the sequence length (i.e., we used 4, 5, and 6 digits) and the number range (i.e., 1–9 except 5 as well as 1–10).

Different hypotheses can be derived according to the visuo-spatial and the verbal–spatial account vs. the working memory account. According to the visuo-spatial and the verbal-spatial account, we should observe a reliable SNARC effect, because we did not use a descending order in the sequence of the to-be-remembered numbers. However, according to the working memory account we would expect the SNARC effect to be reduced or absent at all, because the sequence of the to-be-remembered numbers in working memory does not follow the inherent ordinal structure of the number system.

Neither of the accounts can explain a modulation of the SNARC effect by sequence length. However, Ginsburg and Gevers (2015) suggested that long-term memory associations between number magnitude and space as well as temporarily established associations between the ordinal position of a number and space coexist in parallel. Thus, it might be possible that although spatial-numerical associations are stored in long-term memory, they might nevertheless be also activated in working memory temporarily during a parity judgment task. Accordingly, an influence of sequence length on the SNARC effect should be expected. This notion would also explain the diverging results of Lindemann et al. (2008) vs. van Dijck and Fias (2011) as well as Ginsburg et al. (2014). Furthermore, we would expect that the ordinal position effect should be modulated by sequence length because memorizing digits requires working memory resources.

Finally, neither of the accounts offers an answer to the question whether the number range affects the SNARC effect. However, as argued above, inclusion of the number “10” might influence the size of the SNARC effect, which might explain the null effects of van Dijck and Fias (2011) and Ginsburg et al. (2014).

## Materials and Methods

### Participants

Thirty-four students of the University of Tuebingen participated in the study (20 female). Average age was 25.12 years (*SD* = 3.19 years, range: 19–31 years). All but three participants were right-handed. All reported normal or corrected-to-normal vision. The study was approved by the local Ethics Committee of the Leibniz-Institut für Wissensmedien. All subjects gave written informed consent in accordance with the Declaration of Helsinki and were compensated with 20€ for participation in the study.

### Apparatus

Stimuli were presented in white against a black background at the center of the screen (font: Arial, size: 60) on a 14″ Lenovo IdeaPad S400 notebook with resolution set to 1024 × 768 pixels. The experiment was programmed using Presentation software (https://www.neurobs.com/presentation).

### Stimuli, Design, and Procedure

We used two number ranges: (1) numbers ranging from 1 to 4 and from 6 to 9 and 2) numbers ranging from 1 to 10.

**FIGURE 1:**
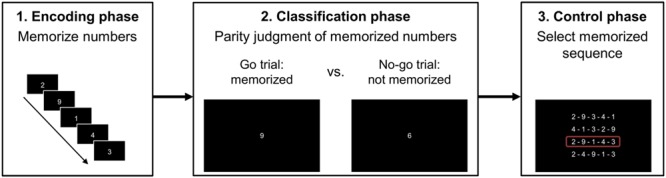
**Procedure for an experimental block**.

At the beginning of the experiment, participants were instructed verbally about the tasks. The experiment was administered in 54 blocks. Each block consisted of an encoding phase, a classification phase, and a control phase (cf. **Figure 1**).

In the encoding phase, participants had to memorize a sequence of either four, five, or six numbers presented individually. We generated 3 (four, five, or six memorized numbers) × 8 sequences for the number range 1–9 and 3 (four, five, or six memorized numbers) × 10 sequences for the number range 1–10 such that each number appeared at each position of the memorized sequence once (see **Table 1**). Memorizing of the sequence was self-paced. After pressing the enter key, the screen turned black for 500 ms followed by the next number of the sequence.

**Table 1:** Sequences employed in the present study.

	Sequence length		
Number range	4	5	6
1–9 (except 5)	4–1–8–7	6–9–1–7–4	7–2–8–3–6–1
	3–9–1–8	8–2–6–3–7	4–9–1–8–3–7
	1–6–3–4	9–3–7–2–6	3–7–2–6–1–4
	8–2–7–3	3–7–4–9–1	9–1–4–2–8–6
	7–8–2–6	1–8–3–4–2	2–8–6–7–4–9
	6–4–9–1	7–4–9–1–8	6–3–9–4–7–2
	9–3–4–2	2–1–8–6–9	1–4–3–9–2–8
	2–7–6–9	4–6–2–8–3	8–6–7–1–9–3
1–10	3–8–5–7	2–9–6–8–3	3–7–4–5–2–6
	4–5–1–6	5–10–8–9–1	5–4–7–1–10–3
	7–1–8–4	7–8–4–5–2	9–1–3–2–8–5
	9–6–7–5	9–3–5–4–8	8–3–5–4–6–1
	1–10–3–9	4–2–9–1–5	1–6–2–7–4–10
	6–7–2–10	10–4–7–3–6	7–10–6–9–1–2
	10–4–9–2	1–5–2–10–7	6–9–8–10–5–7
	8–2–10–3	6–1–3–2–10	10–8–9–3–7–4
	5–3–6–1	3–6–1–7–4	2–5–1–8–3–9
	2–9–4–8	8–7–10–6–9	4–2–10–6–9–8

After rehearsal for 2,500 ms, the classification phase started, in which participants had to classify the parity status of numbers by pressing either the left or the right Ctrl key of a standard QWERTZ keyboard. All of the 8 (range 1–9 except 5) or 10 (range 1–10) different numbers were presented twice in random order (go as well as no-go trials). Importantly, participants only had to press a respective response key, when a number of the to-be-memorized sequence was presented (go trial). Otherwise, they should not press any key (no-go trial). A white fixation cross was presented for 500 ms (font: Arial, size: 60) to indicate separation of trials.

Finally, in the control phase, four different sequences of four, five, or six numbers were presented, from which participants had to indicate the to-be-memorized set, by pressing one of the keys 1, 2, 3, or 4 on the keyboard (upper row of the keyboard). Incorrect sequences were random permutations of the correct sequence. Similar to van Dijck and Fias (2011), we aimed at investigating whether participants memorized the sequence correctly. In case participants did not pick the correct sequence, they had to complete the block containing the respective sequence again at a later stage of the experiment. After participants had chosen a sequence, the next block started.

The participants did not get feedback in any of the phases. Moreover, the experiment was subdivided into two parts. In one part, participants had to press the left key for odd numbers and the right key for even numbers, whereas the hand-to-response assignment was reversed in the other half. The order of hand-to-response assignments was counterbalanced across participants. In total, participants had to complete at least 108 blocks (54 × 2) consisting of 60 × 10 × 2 (range 1–10) + 48 × 8 × 2 (range 1–9 except 5) = 1968 trials.

Furthermore, we administered the experiment in two sessions on two different days, because participants needed about 1 or 1.5 h(s) to complete the 24 (number range 1–9) or 30 sequences (number rage 1–10) consisting of either 48 or 60 blocks, respectively. In total, the experiment took about 2.5 h. In one of the sessions, participants completed sequences containing either the number range 1–9 or the number range 1–10. The order of the sessions was counterbalanced across participants, such that half of the participants started with the number range 1–9 and the other half started with the number range 1–10.

### Analysis

Only go-trials from blocks, for which the to-be-memorized sequence was identified correctly, were considered for RT analyses. Thus, we excluded about 2.26 blocks and about 40.58 trials (go as well as no-go trials) per participant. We included only correctly solved trials (89.5% of all go-trials). Moreover, we excluded correct RTs outside the interval ± 3SD around the individual mean (0.02% of all trials). In sum, we considered 89.2% of all trials in the RT analysis.

We manipulated the following factors in our study: number magnitude (1–9 except 5 or 1–10, respectively), position of target number in memorized sequence (1–4, 1–5, or 1–6, respectively), response side (left hand vs. right hand), sequence length (4–6, i.e., the number of digits, which had to be memorized), and number range (1–9 except 5 or 1–10, respectively). The SNARC effect is indicated by an interaction between number magnitude and response side while the ordinal position effect is reflected by an interaction between position and response side.

In the present study, we were primarily interested in interactions between the SNARC/ordinal position effect and sequence length and/or number range. Accordingly, we first estimated SNARC and ordinal position effects for each participant separately using the by-participant regression approach (Lorch and Myers, 1990; Fias et al., 1996). Thus, to estimate the SNARC effects we ran linear regressions for RTs as well as error rates including number magnitude and response side as predictor variables for each sequence length (4–6), number range (1–9 except 5 or 1–10, respectively), and position in memorized sequence (initial vs. final) separately. We divided the positions of target numbers within the to-be-memorized sequence into two groups to increase the number of data points in the regression, because we collected only two data points per number magnitude, position, response side, sequence length, number range and participant. These data points were further reduced due to missing and erroneous responses. Dividing the positions into two groups increased the number of potential data points to four data points per digit ensuring that we had enough data points to estimate the SNARC slopes. We coded trials according to their position in to-be-memorized sequences into initial elements when the respective digit occurred at position 1 or 2 for sequence lengths 4 and 5, and 1 to 3 for sequence length 6. Likewise, final elements of the memorized sequences were those digits occurring at position 3 to 4 for sequence length 4, 4 to 5 for sequence length 5, and 4 to 6 for sequence length 6. We did not consider position 3 of sequence length 5, because it could be neither categorized as initial or final element.

Similarly, to estimate ordinal position effects we ran linear regressions for RT data including position of target number in to-be-memorized memorized sequence and response side as predictor variables for each sequence length (4–6), number range (1–9 except 5 or 1–10) and number magnitude (small vs. large), respectively. Again, we increased the number of data points in the regression analyses by pooling number magnitude into two groups. Numbers 1–4 (number range 1–9 except 5) and numbers 1–5 (number range 1–10) were coded as small, and numbers 6–9 (number range 1–9 except 5) and numbers 5–10 (number range 1–10) were coded as large.

The outcome of these regression analyses were slope estimates for the SNARC as well as the ordinal position effect. As it is common practice in research on the SNARC effect, we coded response side in the regression analyses such that negative slopes indicated relatively faster responses with the left hand (left response side) to small numbers/initial elements and faster responses with the right hand (right response side) to large numbers/last elements.

In the next step, we evaluated whether slope estimates differed between sequence lengths, number ranges, and position in memorized sequence or number magnitude, respectively. Therefore, we ran two ANOVAs, one with the SNARC effect and one with the ordinal position effect as dependent variable. In the ANOVA for the SNARC effect, we included sequence length (4–6), number range (1–9 except 5 or 1–10, respectively), and position in memorized sequence (initial vs. final) as factors, whereas in the ANOVA for the ordinal position effect, we included sequence length (4–6), number range (1–9 except 5 or 1–10, respectively), and number magnitude (small vs. large) as factors.

Statistical analyses were carried out using R (R Core Team, 2015) and the R package afex for running ANOVA (Singmann et al., 2015). Furthermore, to quantify the probability in favor of the null-hypothesis, we ran Bayesian analyses using the R package Bayes Factor (Morey and Rouder, 2015).

## Results

An overview of SNARC effects and ordinal position effects in the respective conditions is given in **Tables 2** and **3** (RT/error rates). Moreover, a summary of ANOVA results is given in **Table 4**.

**Table 2:** Means (SD in parenthesis) and results of one sample *t*-tests against zero for SNARC and ordinal position effects (RTs in ms) for different sequence lengths, number ranges, and positions in memorized sequence or number magnitude, respectively.

Number range	Sequence length	Position	*M* (*SD*)	*t*	*p*
**SNARC effect**					
1–9	4	Initial	–9.33 (19.18)	–2.84	0.008
		Last	–8.51 (17.27)	–2.87	0.007
	5	Initial	–9.84 (13.43)	–4.27	<0.001
		Last	–9.55 (17.14)	–3.25	0.003
	6	Initial	–7.87 (19.11)	–2.40	0.022
		Last	–6.91 (16.84)	–2.39	0.023
1–10	4	Initial	–2.36 (14.13)	–0.98	0.336
		Last	–5.44 (16.10)	–1.97	0.057
	5	Initial	–5.44 (17.93)	–1.77	0.086
		Last	–5.10 (15.96)	–1.86	0.071
	6	Initial	–6.50 (15.49)	–2.45	0.020
		Last	–3.11 (17.23)	–1.05	0.301
**Ordinal position effect**					
1–9	4	Small	–24.21 (46.70)	–3.02	0.005
		Large	–5.51 (43.63)	–0.74	0.466
	5	Small	–7.30 (33.09)	–1.29	0.207
		Large	–8.28 (30.14)	–1.60	0.119
	6	Small	–5.11 (24.63)	–1.21	0.235
		Large	–5.90 (24.34)	–1.41	0.167
1–10	4	Small	–7.67 (48.82)	–0.92	0.366
		Large	–15.86 (36.89)	–2.51	0.017
	5	Small	–10.60 (32.94)	–1.88	0.070
		Large	–8.30 (24.60)	–1.97	0.058
	6	Small	–12.10 (27.50)	–2.57	0.015
		Large	–1.38 (22.26)	–0.36	0.720

*SNARC/ordinal position effect slopes were computed for dRT = RT right hand – RT left hand. Thus, negative slopes indicate faster responses for small numbers/initial elements with the left hand and faster responses for large numbers/last elements with the right hand.*

**Table 3:** Means (SD in parenthesis) and results of one sample *t*-tests against zero for SNARC and ordinal position effects (error rates in %) for different sequence lengths, number ranges, and positions in the memorized sequence or number magnitude, respectively.

Number range	Sequence length	Position	*M* (*SD*)	*t*	*p*
**SNARC effect**					
1–9	4	Initial	–0.89% (2.91%)	–1.79	0.082
		Last	–0.74% (3.97%)	–1.08	0.288
	5	Initial	–0.76% (2.89%)	–1.53	0.135
		Last	–0.89% (2.46%)	–2.12	0.041
	6	Initial	–1.58% (2.63%)	–3.50	0.001
		Last	–0.70% (2.37%)	–1.73	0.093
1–10	4	Initial	–1.32% (2.24%)	–3.44	0.002
		Last	–0.31% (2.41%)	–0.74	0.463
	5	Initial	–0.12% (1.99%)	–0.37	0.717
		Last	–0.43% (2.77%)	–0.90	0.374
	6	Initial	–0.97% (2.31%)	–2.44	0.020
		Last	–0.97% (3.08%)	–1.83	0.077
**Ordinal position effect**					
1–9	4	Small	–3.38% (6.05%)	–3.26	0.003
		Large	–2.61% (8.19%)	–1.86	0.072
	5	Small	–1.65% (4.16%)	–2.32	0.027
		Large	–2.72% (4.15%)	–3.83	<0.001
	6	Small	–0.64% (4.22%)	–0.89	0.382
		Large	–0.14% (4.17%)	–0.19	0.850
1–10	4	Small	–3.27% (5.76%)	–3.31	0.002
		Large	–0.83% (6.04%)	–0.80	0.427
	5	Small	–0.81% (6.56%)	–0.72	0.477
		Large	–0.81% (3.70%)	–1.27	0.211
	6	Small	–1.14% (3.82%)	–1.75	0.090
		Large	–0.64% (2.81%)	–1.32	0.195

*SNARC/ordinal position effect slopes were computed for dRT = RT right hand – RT left hand. Thus, negative slopes indicate faster responses for small numbers/initial elements with left hand and faster responses for large numbers/last elements with right hand.*

**Table 4:** ANOVA results for SNARC effect and ordinal position effect.

DV	Effect	*df1*	*df2*	*F*	*p*
SNARC (RT)	Intercept	1	33	32.01	<0.001
	Sequence	2	66	0.26	0.774
	Range	1	33	7.11	0.012
	Position	1	33	0.09	0.772
	Sequence × Range	2	66	0.23	0.799
	Sequence × Position	2	66	0.36	0.697
	Range × Position	1	33	0.02	0.887
	Sequence × Range × Position	2	66	0.36	0.698
Ordinal position (RT)	Intercept	1	33	18.38	<0.001
	Sequence	2	66	1.83	0.169
	Range	1	33	<0.01	0.985
	Position	1	33	1.48	0.232
	Sequence × Range	2	66	0.17	0.847
	Sequence × Position	2	66	0.24	0.790
	Range × Position	1	33	0.47	0.496
	Sequence × Range × Position	2	66	2.99	0.057
SNARC (ER)	Intercept	1	33	17.92	<0.001
	Sequence	2	66	1.09	0.341
	Range	1	33	0.76	0.389
	Position	1	33	0.91	0.348
	Sequence × Range	2	66	0.49	0.617
	Sequence × Position	2	66	1.08	0.344
	Range × Position	1	33	0.01	0.914
	Sequence × Range × Position	2	66	1.17	0.318
Ordinal position (ER)	Intercept	1	33	31.75	<0.001
	Sequence	2	66	6.3	0.003
	Range	1	33	1.97	0.169
	Position	1	33	0.58	0.450
	Sequence × Range	2	66	1.16	0.321
	Sequence × Position	2	66	1.33	0.272
	Range × Position	1	33	0.81	0.376
	Sequence × Range × Position	2	66	0.28	0.758

*Resp. side = Response side, Sequence = Sequence length, and Range = Number Range.*

### Response Times

First, we examined whether SNARC effects differed between sequence lengths, number ranges, and positions running a repeated measures ANOVA with the SNARC effect estimate as dependent variable. The ANOVA revealed that the intercept was significantly different from zero (see **Table 4**) indicating that we found a significant SNARC effect across all conditions (*M* = –6.66 ms, *SD* = 6.87 ms, 95% CI = [–9.06 ms, –4.27 ms]). Moreover, we observed a significant main effect of number range (see **Table 4**). The SNARC effect was larger when number range 1–9 (*M* = –8.67 ms, *SD* = 7.14 ms, 95% CI = [–11.47 ms, –5.87 ms]) was employed than when number range 1–10 was employed (*M* = –4.66 ms, *SD* = 9.04 ms, 95% CI = [–7.46 ms, –1.86 ms]).

Furthermore, we also calculated the probability in favor of the null hypothesis for the factor sequence length. The posterior probability for the null hypothesis was *P*(*H_0_*|*D*) = 0.77. According to a suggestion by Raftery (1999), this indicates positive evidence in favor of the null hypothesis suggesting that SNARC effects did not differ between different sequence lengths.

Second, we investigated the influence of sequence length, number range, and magnitude on the ordinal position effect by running a repeated measures ANOVA with the ordinal position effect estimate as dependent variable. Again, the intercept was significantly different from zero indicating a significant position effect across all conditions (*M* = –9.35 ms, *SD* = 12.72 ms, 95% CI = [–13.79 ms, –4.91 ms]). As can be seen in **Table 3**, no main effect or interaction was significant.

**FIGURE 2:**
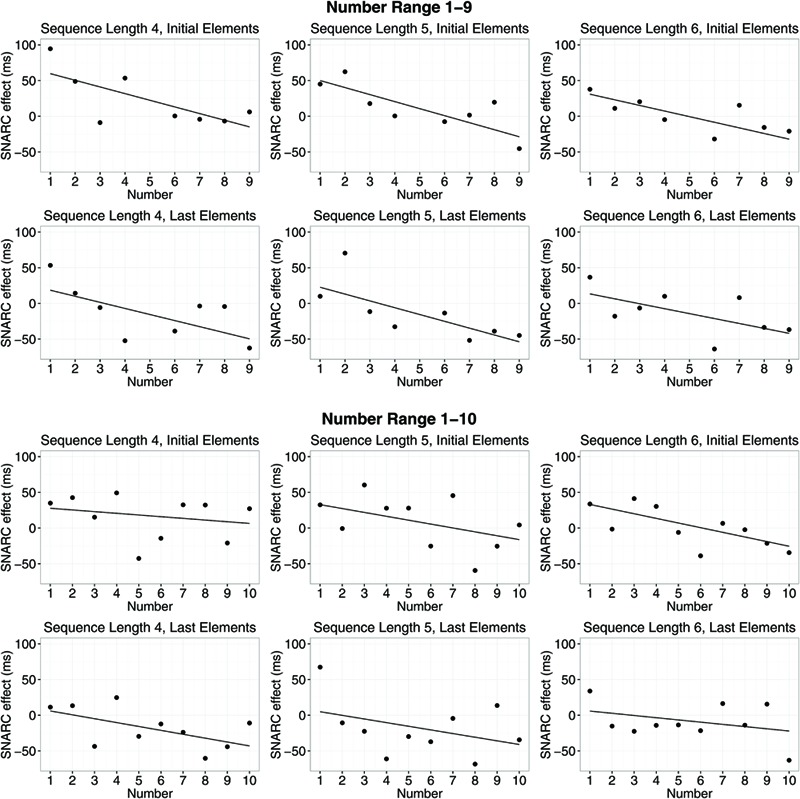
**Spatial-numerical association of response codes (SNARC) effects (response times, RT) for different number ranges, sequence lengths, and positions in the sequence of memorized numbers.** Dots show average RT differences of right hand minus left hand RTs. Lines indicate means of slope estimates for the interaction between number magnitude and response side (i.e., the SNARC effect).

**FIGURE 3:**
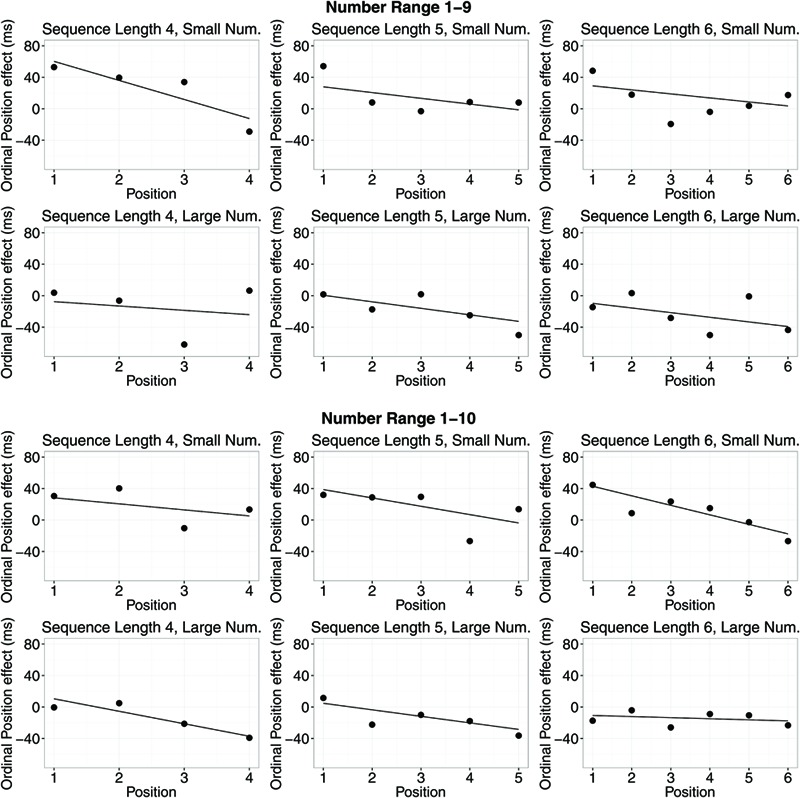
**Ordinal position effects (RT) for different number ranges, sequence lengths, and small/large number magnitudes.** Dots show average RT differences of right hand minus left hand RTs. Lines indicate means of slope estimates for the interaction between position in the memorized sequence and response side (i.e., the ordinal position effect).

In **Figures 2** and **3**, SNARC effects and ordinal position effects (RT) for different number ranges, sequence lengths, and positions in the sequence of memorized numbers are depicted.

### Error Rates

The analyses for RT data were also conducted identically for error rates. The ANOVA with the SNARC effect as dependent variable revealed that the intercept was significantly different from zero (see **Table 4**) indicating a significant SNARC effect across conditions (*M* = –0.81%, *SD* = 1.11%, 95% CI = [–1.19%, –0.42%]). In contrast to RT data, we did not observe a main effect for condition. Moreover, no other main effect or interaction was significant. Again, we ran a Bayesian analysis to calculate the probability in evidence of the null hypothesis for the factor sequence length. The posterior probability for the null hypothesis was *P*(*H_0_*|*D*) = 0.71. According to the operational definition provided by Raftery (1999), this indicates only weak evidence in favor of the null hypothesis (i.e., no effect of sequence length on the SNARC effect).

For the ordinal position effect, the ANOVA also revealed that the intercept differed significantly from zero (see **Table 4**) indicating a significant ordinal position effect across conditions (*M* = –1.55%, *SD* = 1.61%, 95% CI = [–2.12%, –0.99%]). Moreover, the main effect of sequence length was significant (see **Table 3**). The ordinal position effect decreased with increasing numbers which had to be memorized. The ordinal position effect was for sequence length 4, 5, and 6: –2.52% (*SD* = 2.92%), –1.50% (*SD* = 2.03%), and –0.64% (*SD* = 2.18%). *Post-hoc* tests revealed that only ordinal position effects for sequence length 4 and 6 differed significantly from each other (*p* = 0.002).

**FIGURE 4:**
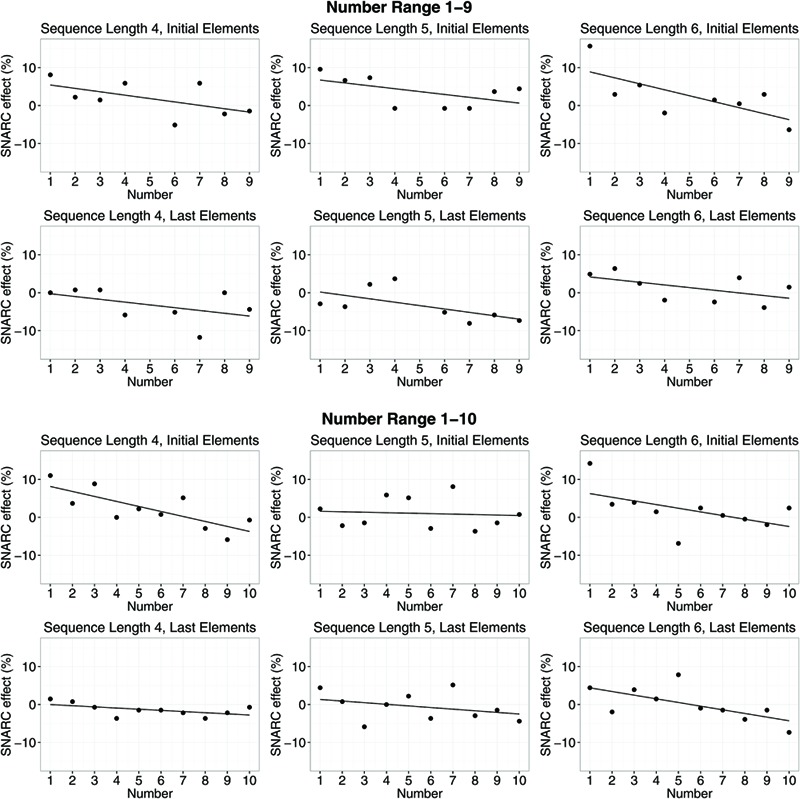
**SNARC effects (error rates) for different number ranges, sequence lengths, and positions in the sequence of memorized numbers.** Dots show differences in error rates for right hand minus left hand. Lines indicate means of slope estimates for the interaction between number magnitude and response side (i.e., the SNARC effect).

**FIGURE 5:**
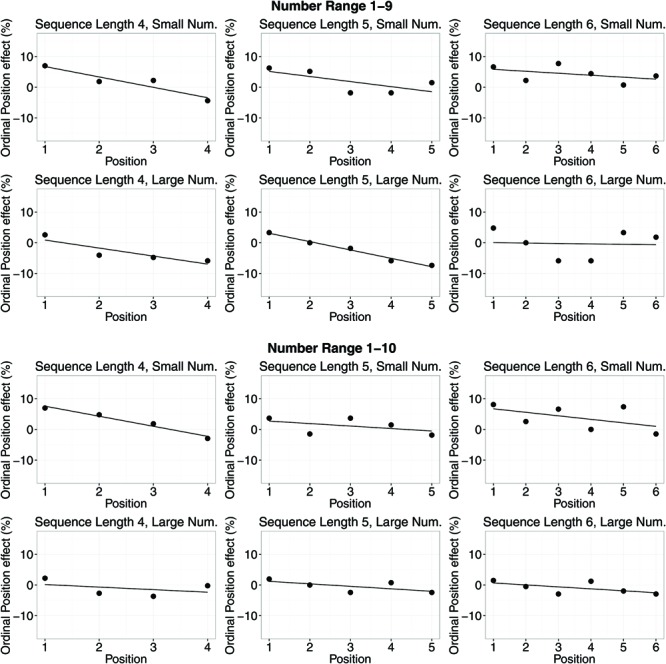
**Ordinal position effects (error rates) for different number ranges, sequence lengths, and small/large number magnitudes.** Dots show differences in error rates for right hand minus left hand. Lines indicate means of slope estimates for the interaction between position in the memorized sequence and response side, i.e., the ordinal position effect.

In **Figures 4** and **5**, SNARC effects and ordinal position effects (error rates) for different number ranges, sequence lengths and positions in the sequence of memorized numbers are shown.

## Discussion

We evaluated the relationship between the SNARC and the ordinal position effect. According to the working memory account for the SNARC effect, the SNARC effect originates from an ordinal position effect (Ginsburg et al., 2014). While performing a parity judgment task, participants activate the respective number range from which the to-be-evaluated numbers are taken in working memory according to their ordinal structure (e.g., 1, 2, 3, 4, etc.). Initial elements of this list are then associated with left response side and last elements with right response side. In case participants have to memorize numbers in random order, they cannot make use of the ordinal structure of numbers and hence, the SNARC effect should be reduced or even disappear. However, this interpretation of the SNARC effect is at odds with the findings of Lindemann et al. (2008) who observed a regular SNARC effect although participants had to memorize numbers in randomized order.

We evaluated two possible explanations for this divergence of results from the study by Ginsburg et al. (2014) as well as van Dijck and Fias (2011), who did not observe a SNARC effect when participants had to memorize numbers presented in random order: (1) the length of the sequence of to-be-memorized numbers and (2) the number range employed. In the study of Lindemann et al. (2008), participants had to memorize three numbers, whereas in the study of Ginsburg et al. (2014) as well as van Dijck and Fias (2011), they had to memorize five numbers. Moreover, numbers ranged from 1 to 9 (except 5) in the study of Lindemann et al. (2008), whereas number ranged from 1 to 10 in the study of Ginsburg et al. (2014) as well as van Dijck and Fias (2011).

To evaluate these two possible explanations, we manipulated the length of the sequence of to-be-memorized numbers from 4 to 6 and employed both number ranges, i.e., 1–9 (except 5) and 1–10. Our results indicated that sequence length had no impact on the SNARC effect, but we found that number range modulated the size of the SNARC effect. The SNARC effect was smaller for number range 1–10 than for number range 1–9. Thus, our results suggest that the null SNARC effect found in the studies by van Dijck and Fias (2011) as well as Ginsburg et al. (2014) might be caused by the different number ranges employed. The number 10 is not only problematic because it is a two-digit number but also because the leading odd digit 1 may well have interfered with the “even” response for 0 (Tan and Dixon, 2011; Huber et al., 2015). Furthermore, the parity status of zero differs from that of other even digits as participants are often not sure about it (e.g., Nuerk et al., 2004). In sum, the results of the present study support the notion that including the number 10 in the stimulus set seems to reduce the SNARC effect in parity judgment tasks (e.g., Nuerk et al., 2004).

Moreover, the finding of a significant SNARC effect contradicts the exclusive working memory account, according to which the SNARC effect should be reduced or disappear when participants have to memorize numbers in working memory in a random or reversed numerical order. Thus, our results support the idea that both long-term memory associations between number magnitude and space as well as temporarily established associations between the ordinal position of a number and space coexist in parallel (Ginsburg and Gevers, 2015). Consequently, spatial-numerical and spatial-positional associations are not mutually exclusive in a parity judgment task. This view corresponds to the suggestion of Ginsburg and Gevers (2015), who supposed that the SNARC effect can be explained in terms of activations in long-term memory, whereas the ordinal position effect originates from temporarily created bindings of elements in a spatial order in working memory.

However, our results do not indicate that SNARC effects cannot be overwritten by holding a sequence of numbers in working memory. In the present study, participants had to memorize an unordered sequence of numbers. As in the study by Lindemann et al. (2008), we observed a significant SNARC effect. However, Lindemann et al. (2008) also observed that the SNARC effect diminishes in case of descending order. Thus, it seems that descending sequences are critical for the observation of a reduced/absent SNARC effect (i.e., the default ordinal structure of numbers stored in long-term memory). Evidence for this suggestion comes from several studies which did not find a significant SNARC effect in a particular condition with a reversed sequence of the numbers. For instance, Bächtold et al. (1998) presented numbers on a clock face with small digits being on the right side and large digits on the left side (i.e., they employed a descending order), and Fischer et al. (2010) reordered digits by presenting large digits on the left or small digits on the right side of text (i.e., again a descending order was employed). In both cases a SNARC effect was absent or even reversed.

Furthermore, we found that – in contrast to the SNARC effect – the ordinal position effect for error rates was modulated by working memory load as reflected by a reduction of that effect, the more numbers had to be memorized. This finding suggests that whether the spatial coding of elements in working memory influences performance depends on working memory load and that the ordinal position effect arises from WM encoding (e.g., Ginsburg et al., 2014).

However, there are some limitations to the present study that need to be acknowledged when interpreting these data. In the present study, we had few repetitions per digit (see Cipora and Nuerk, 2013, for the negative effect of a small number of repetitions on the SNARC effect). Therefore, we clustered positions in the memorized sequence into initial and last elements resulting in at least four data points per number and response hand. Nevertheless, this potential limitation might have reduced the possibility to find an interaction of the SNARC effect with sequence length. To address this concern, we ran a Bayesian analysis and found positive evidence for a null effect in the RT data. Moreover, even with only four repetitions per number, we were able to observe significant SNARC effects for RT data and number range 1-9 irrespective of sequence length. However, the small number of repetitions might have affected our findings for error rates, because we did not observe an interaction between the SNARC effect and number range for error rates. Moreover, the Bayesian analysis suggested that there was only weak evidence in favor of the null hypothesis. Thus, whether or not the SNARC effect for error rates is also affected by number range, has to be addressed in a further study employing a larger number of repetitions to increase the reliability of the SNARC effect. However, our study already lasted for about 2.5 hours. Increasing the number of repetitions would further prolong the experiment. Hence, a critical issue in such a study would be keeping the participants motivated and controlling for adverse effects of fatigue.

## Conclusion

The present study provides evidence against an exclusive working memory account supporting the idea that long-term memory associations between number and space exist independent of temporary associations or ordinal positions in working memory.

## Author Contributions

Conceived and designed the experiments: SH, EK, KM, KW. Analyzed the data: SH. Wrote the paper: SH, EK, KM, KW.

## Conflict of Interest Statement

The authors declare that the research was conducted in the absence of any commercial or financial relationships that could be construed as a potential conflict of interest.
